# Iron Status in Attention-Deficit/Hyperactivity Disorder: A Systematic Review and Meta-Analysis

**DOI:** 10.1371/journal.pone.0169145

**Published:** 2017-01-03

**Authors:** Yan Wang, Lan Huang, Li Zhang, Yi Qu, Dezhi Mu

**Affiliations:** 1 Department of Pediatrics, West China Second University Hospital, Sichuan University, Chengdu, China; 2 Key Laboratory of Birth Defects and Related Diseases of Women and Children (Sichuan University), Ministry of Education, Chengdu, China; 3 Department of Pediatrics, University of California, San Francisco, California, United States of America; Pennsylvania State University College of Medicine, UNITED STATES

## Abstract

**Background:**

Attention-deficit/hyperactivity disorder (ADHD) is one of the most common psychiatric disorders in children. However, the pathogenesis of ADHD remains unclear. Iron, an important trace element, is implicated in brain function and dopaminergic activity. Recent studies have investigated the association between iron deficiency and ADHD, but the results are inconsistent.

**Methods:**

A systemic search of MEDLINE, EMBASE, Web of Science and Cochrane Library databases was supplemented by manual searches of references of key retrieved articles. Study quality was evaluated using the Newcastle-Ottawa Scale. The standardised mean difference (SMD) and 95% confidence intervals (CIs) were calculated using a random-effects model. H^2^ and I^2^ were used to evaluate the heterogeneity, and sensitivity, subgroup and meta-regression analyses were conducted to explore the reason of heterogeneity.

**Results:**

The search yielded 11 studies published before July 25, 2016. Of these, 10 studies, comprising 2191 participants and 1196 ADHD cases, reported serum ferritin levels, and six studies, comprising 617 participants and 369 ADHD cases, reported serum iron levels. Serum ferritin levels were lower in ADHD cases (SMD = -0.40, 95% CI = -0.66 to -0.14). However, we found no correlation between serum iron levels and ADHD (SMD = -0.026, 95% CI = -0.29 to 0.24). Meta-regression analysis indicated that publication year, age, gender, sample size, and Hb levels did not significantly influence the pooled estimates of serum ferritin.

**Conclusion:**

Lower serum ferritin rather than serum iron is associated with ADHD in children.

## Introduction

As one of the most common childhood psychiatric disorders, attention-deficit/hyperactivity disorder (ADHD) is estimated to affect approximately 5.9–7.1% of children and adolescents worldwide [1]. Inattention, impulsivity, and/or hyperactive/impulsive behaviors are key features of ADHD. It has also been associated with other psychiatric comorbidities, such as disruptive behaviors, antisocial activities, and poor verbal working memory disorders, resulting in impaired learning and social ability [2, 3]. ADHD is commonly considered to develop as a result of interactions between genetic and environmental factors [4]. However, the specific underlying etiology of ADHD remains unclear.

Several studies of the neurobiology and treatment of ADHD have suggested that nutritional factors, such as glucose metabolism, fatty acid metabolism, and mineral or vitamin deficiencies, may affect brain function and are implicated in the pathogenesis of the disorder [5–7]. Of these nutritional factors, iron deficiency has attracted more attention as iron plays an important role in the regulation of dopaminergic activity, which is associated with the pathogenesis and symptoms of ADHD [8].

In 1997, Sever et al. [9] found significantly increased serum ferritin levels and decreased ADHD symptom scores in children with ADHD after iron supplementation, suggesting that nonanemic children with ADHD may benefit from iron supplementation. Subsequently, other researchers attempted to explore the relationship between iron deficiency and ADHD. However, their conclusions were often inconsistent. Some authors reported that mean serum ferritin levels were lower in children with ADHD than in healthy controls [8, 10, 11]. Furthermore, Juneja et al. [10] reported that serum ferritin levels were inversely correlated with ADHD symptom severity. Moreover, Cortese et al. [8] compared brain iron levels in children with ADHD and healthy controls using magnetic resonance imaging (MRI), reporting significantly lower estimated brain iron in the bilateral thalami of children with ADHD. Similarly, Adisetiyo et al. [11] found that medication-naïve patients with ADHD had lower estimated brain iron in the striatum and thalamus than did healthy controls. However, other authors have failed to find a relationship between serum ferritin and ADHD [12–14].

In 2012, Cortese et al. [15] published a systematic review of studies investigating the correlation between iron and ADHD. However, they did not perform statistical analyses on indices of iron status. Moreover, several studies with large sample sizes have been published after 2012. We therefore conducted a systematic review and meta-analysis, including more recent data, to estimate the association between iron status and ADHD.

## Materials and Methods

### Study Retrieval

We searched PubMed, EMBASE, Web of Science and Cochrane Library from inception to July 25, 2016. The search strategy combined Medical Subject Heading (MeSH) terms and free text terms, including ‘‘attention-deficit/hyperactivity disorder” or ‘‘ADHD” or “attention deficit disorders with hyperactivity” or ‘‘hyperkinetic disorder” or “hyperkinetic syndrome” and “iron” or “siderophilin” or “serotransferrin” or “transferrin” or “ferritin” or “iron deficiency anemia” or “ID.” We only included studies on humans published in English, but study location was not restricted. Additionally, the references of included studies and previous reviews were screened manually to identify additional appropriate studies for inclusion.

### Study Selection

Two investigators independently selected the studies. Initially, clearly irrelevant studies were excluded by scanning titles and abstracts. The full texts of the remaining articles were then evaluated carefully according to our eligibility criteria. Where required, any disagreement on eligibility for inclusion was resolved by a third author.

We included studies that met all the following criteria: 1) they assessed serum ferritin or serum iron in children or adults with ADHD and non-ADHD controls; 2) patients with ADHD were diagnosed according to formal criteria (e.g. the Diagnostic Statistical Manual of Mental Disorders [DSM]-IV [TR] or previous versions); 3) they provided the mean and standard deviation for raw data. We excluded case reports, conference abstracts, reviews, and animal studies. Studies without raw data or reporting iron status in tissues other than the blood were also excluded.

### Data Extraction

Two reviewers independently extracted information on the first author, publication year, country, study design, exclusion criteria, age (mean±SD), number of cases and controls, proportion of female and male participants, analytical technology, ferritin (mean±SD), serum iron (mean±SD), and units of measure.

### Quality Assessment

Study quality was rated by two authors using the Newcastle-Ottawa Scale, which is recommended for quality assessment of cohort and case-control studies, and has a maximum score of nine. Studies scoring 7 to 9, 4 to 6, and 0 to 3 are regarded as high quality, moderate quality, and low quality, respectively.

### Statistical Analysis

The standardised mean difference (SMD) was used to assess the association between blood iron status and ADHD. Due to the high heterogeneity between studies, we chose a random-effects model to calculate the pooled SMD. Heterogeneity across studies was assessed using H^2^ [16] and I^2^ [17]. H^2^>1 or I^2^ >50% indicated high heterogeneity. We also used Galbraith radial plots to explore which studies may be contributing to heterogeneity. Additionally, we carried out sensitivity analyses excluding studies that might contribute to high heterogeneity indicated by the Galbraith radial plot.

Subgroup analyses stratified studies by treatment, comorbidities, and assay method. Publication bias was assessed by visual inspection of funnel plots and Egger’s tests. For Egger’s tests, P <0.05 was considered statistically significant. We also performed unrestricted maximum likelihood random-effects meta-regressions, including publication year, mean age, gender (% female), and sample size as regressors. All statistical analyses were performed using Stata 14.0 (Stata Corp, College Station, Texas, USA)

## Results

### Literature Search and Selection

**Fig 1:**
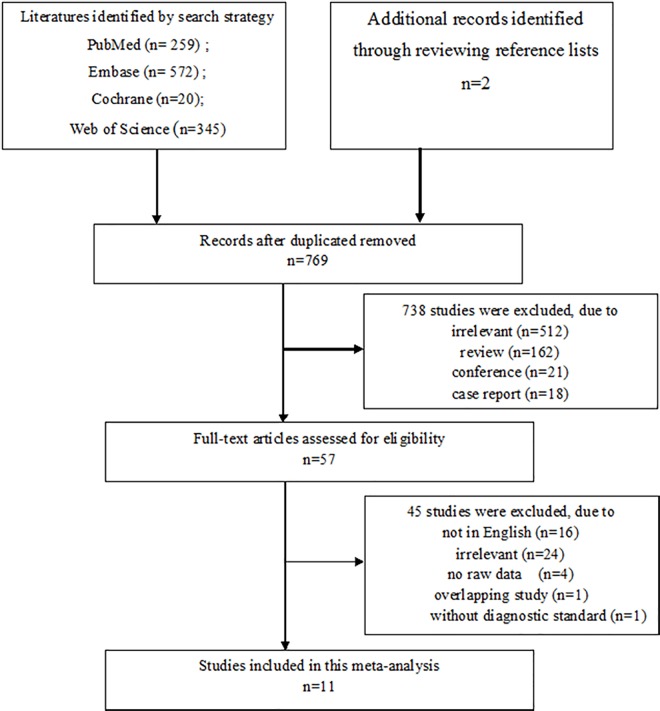
The flow diagram of the selection process.

We screened a total of 1198 articles from PubMed, EMBASE, Cochrane Database, and Web of Science, as well as 2 additional articles from manual searches of the references of articles. After removing 427 duplicates, 162 reviews, 512 irrelevant studies, 18 case reports, and 21 conference abstracts, we retained 57 studies for full-text review. After further screening, 45 studies were excluded, including 16 studies not in English, 24 irrelevant studies, 2 studies without raw data, 1 overlapping study and 1 study without a diagnostic standard. Finally, we included 11 studies (Fig 1). Of these, 10 studies, comprising 2191 participants and 1196 ADHD cases, analyzed serum ferritin and 6 studies, comprising 617 participants and 369 ADHD cases, analyzed serum iron.

### Study Characteristics

Table 1 presents the characteristics of the 11 included studies, published from 2004 to 2016. All were case-control studies. The number of participants varied across studies, and ranged from 27 to 1260. Four studies were conducted in Europe [8, 13, 14, 18], one in the USA [11], one in Egypt [19], one in Brazil [20], and four in Asia [10, 21–23]. With the exception of three studies [8, 22, 23], all included studies reported medication use in the patients. As far as we know, psychoeducation as well as drug therapy and/or behavioral intervention are the main strategies for the treatment of ADHD. Drug therapy mainly included stimulants such as such as methylphenidate or atomoxetine, we categorized those patients with ADHD who were medication-naïve or without drug medication for at least one month into the without treatment group.

**Table 1:** Characteristics of included studies.

Author, Year	Area	Total Sample	Total with ADHD	Age Mean±SDADHD Controls	Sex (%M) ADHD Controls	Serum Ferritin and/ or Serum Iron ADHD/Controls	Exclusion Criteria	Psychiatric Comorbid Disorders	Treatment
Chen, 2004	Taiwan	110	58	8.5±2.2; 7.9±2.0	91.4; 76.9	19.7±6.4/16.1±6.8 (iron umol/L)	N/A	N/A	Drug free for 2 months
Konofal, 2004	France	80	53	N/A	84.9; 74.1	23±13/44±22 (ferritin ng/ml)	Additional psychiatric disorders, physical diseases, and malnutrition	No	Drug free for 2 months
Menegassi2010	Brazil	62	41	9.0±2.6 (group1); 8.8±2.4; (group2); 8.9±2.7	80.5; 71.4	56.6±19.2/58.8±28.9(ferritin ng/ml) 79.5±27.1/92±31.4 (iron ug/dl)	IQ<70, psychiatric disorders other than ODD and CD, any medical conditions affecting iron	ODD, CD	Group1 without medication use; Group 2 treated for 3 months
Juneja, 2010	India	50	25	8.44±1.687.96±1.46	84.0; NA	6.04±3.85/48.96±41.64 (ferritin ng/ml)	IQ<85, any chronic illness, or any acute severe illness in last two weeks	ODD	Newly diagnosed
Kwon, 2011	Korea	96	48	6.98±0.397.53±0.63	54.2; 58.3	35.8±16.6/37.1±18.3(ferritin ng/ml) 80.9±33.3/82.0±28.1(iron ug/dl)	IQ<70, psychiatric comorbidities, and neurological disorders	No	Naive
Mahmoud,2011	Egypt	73	58	8.6 ± 1.8; 8.3 ± 1.8	44.8; 48.0	24.8±14.1/32.6±18.7(ferritin ug/dl)	Co-morbid neurological disorders, severely anemia	No	Without medication for 1 month
Cortese, 2011	France	27	18	9.9±1.5; 10.1±2.2	88.9; 55.6	32.4±13.4/51.6±16.4 (ferritin ng/ml)	IQ<70, neurological diseases, comorbid psychiatric disorders expect ODD	ODD	N/A
Donfrance-sco, 2013	Italy	194	101	8.9±2.5; 9.2±3.1	91.1; 88.2	33.0±17.8/33.1±18.7 (ferritin ng/ml)	IQ<70, neurobiological disease, any medical conditions affecting iron	ODD, anxiety, depression, dysthymic disorder	Naive
Adisetiyo,2014	USA	49	22	12.6 ±2.8; 13.3±2.6	68.2; N/A	50.8±25.2/38.2±22.8 (ferritin ng/ml) 76.9±26.0/65.7±28.2 (iron mcg/dl)	Diagnosis of psychotic, major depressive, conduct, tic, or pervasive developmental disorders	Yes	10 medication naïve patients; 12 patients with medication treatment
Bener, 2014	Qatar	1260	630	11.5±3.8; 11.5±3.6	50.0; 49.7	36.3±5.9/38.2±5.6 (ferritin ng/ml) 82.1±13.6/85.6±12.4(iron ng/ml)	Hb <10 g/dL, calcium supplements or vitamin D intake during the last 6 month; epilepsy or antiepileptic drugs	N/A	N/A
Percinel, 2016	Finland	300	200	11.0±2.4; 11.0±3.0	63.5; 60.0	27.9±15.3/30.8±17.5(ferritin ng/ml) 71.9±31/77.9±30.6 (iron ug/dl)	IQ<80, comorbid psychiatric disorder, any medical conditions affecting iron, Hb<12g/L	No	No

N/A not available; IQ intelligence quotient; TIBC total iron binding capacity; ODD oppositional defiant disorder; CD conduct disorder.

According to the Newcastle-Ottawa Scale, the overall methodological quality was good, with 10 studies that were high quality and only one study [18] that was of moderate quality (Table 2).

**Table 2:** The Newcastle-Ottawa scale score of included studies.

Study	Selection	Comparability	Outcome	Total scores
Chen, 2004	3	2	2	7
Konofal, 2004	3	1	2	6
Menegassi, 2010	3	2	2	7
Juneja, 2010	3	2	2	7
Kwon, 2011	4	2	2	8
Mahmoud, 2011	3	2	2	7
Cortese, 2011	4	2	2	8
Donfrancesco, 2013	4	2	3	9
Adisetiyo, 2014	4	2	2	8
Bener, 2014	4	2	2	8
Percinel, 2015	3	2	2	7

### Serum Ferritin and ADHD

**Fig 2:**
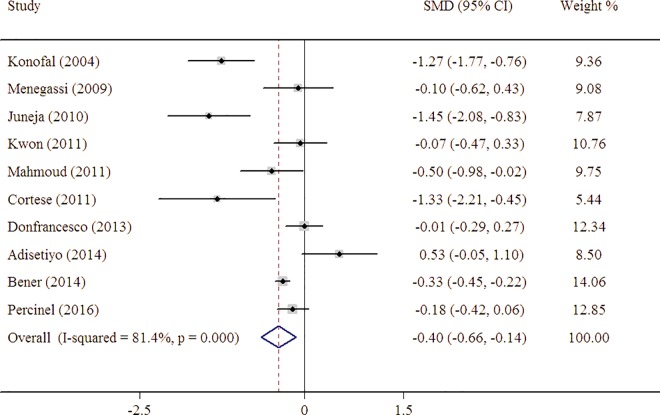
Forest plot for the random-effects meta-analysis of serum ferritin levels in persons with ADHD and controls.

Serum ferritin levels were significantly lower in patients with ADHD compared with healthy controls, with a pooled SMD estimate from 10 studies of -0.40 (95% CI = -0.66 to -0.14) indicating a negative association between serum ferritin and ADHD (Fig 2). However, there was significant statistical heterogeneity across studies (I^2^ = 8.4%, 95% CI 67–90%; H^2^ = 4.37).

### Serum Iron and ADHD

**Fig 3:**
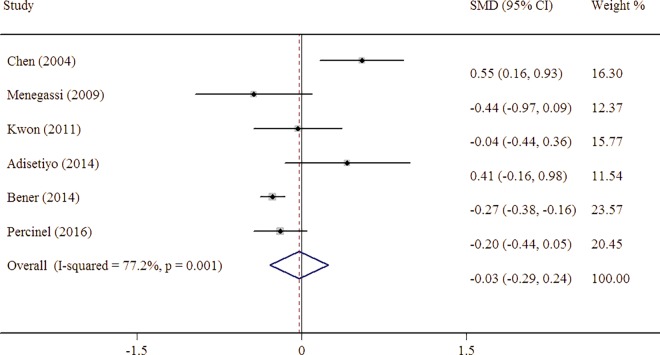
Forest plot for the random-effects meta-analysis of serum iron levels in persons with ADHD and controls.

The pooled estimate from six studies showed a serum iron level of -0.026 (95% CI = -0.29 to 0.24), which did not indicate a negative correlation between serum iron and ADHD (Fig 3). There was statistical heterogeneity across studies with I^2^ 77% (95% CI 49–90%).

### Stratified and Sensitivity Analyses of Serum Ferritin

Of the 10 studies included in the meta-analysis, eight included patients without treatment, with a pooled SMD for serum ferritin of -0.29 (95% CI = -1.26 to 0.68), while patients under treatment had a pooled SMD of -0.36 (95% CI = -0.72 to 0.00). The pooled SMD in five studies of patients with ADHD without other psychiatric disorders was -0.48 (95% CI = -0.94 to -0.01), while the pooled SMD in four studies of patients with ADHD and psychiatric comorbidities was -0.42, (95% CI = -1.06 to 0.22) (Table 3).

**Table 3:** Pooled estimate and heterogeneity in subgroups analyses.

Subgroup analysis	No. of trails	No. of subjects		Pooled estimate (SMD, 95% CI)	Heterogeneity	
		ADHD	Controls		I^2^	P-value
**Treatment**						
Without treatment	8	516	366	-0.36 (-0.72 to 0.00)	82.6	<0.001
Under treatment	3	50	57	-0.29 (-1.26 to 0.68)	80.9	0.005
**Comorbid**						
Yes	5	207	175	-0.42 (-1.06 to 0.22)	82.2	0.001
No	4	359	200	-0.48 (-0.94 to -0.01)	86.6	<0.001
**Assay method**						
ELISA	4	237	170	-0.78 (-1.48 to -0.07)	89.8	<0.001
Others	5	911	787	-0.21 (-0.51 to 0.09)	73.4	0.005

We also conducted a meta-analysis of the assay methods used, separated into ELISA or other methods. The pooled SMD for serum ferritin levels assessed using ELISA was -0.78 (95% CI = -1.48 to -0.07), while for other methods it was -0.21 (95% CI = -0.51 to 0.09).

**Fig 4:**
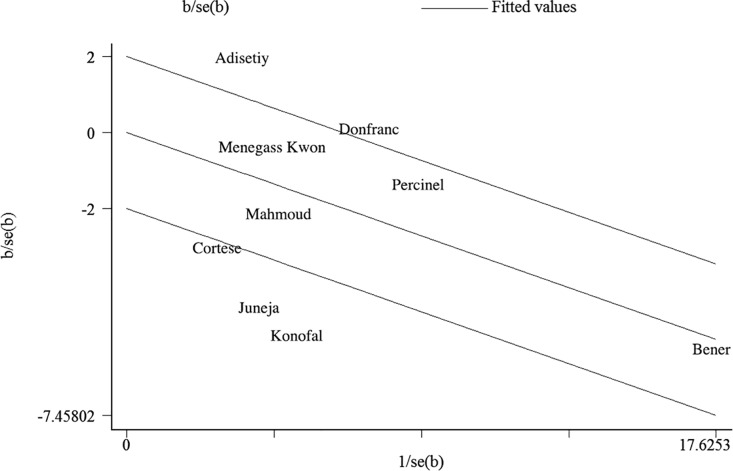
Galbraith radial plot graph of included studies.

The Galbraith radial plot graph (Fig 4) showed four studies [8, 10, 11, 18] that might contribute to the high heterogeneity across studies. Further sensitivity analysis indicated that the overall pooled results did not vary substantially after omitting each of the four studies one at a time (Table 4).

**Table 4:** The sensibility analysis of included studies for serum ferritin.

Study omitted	Estimate	95% Confidence Interval	
Konofal (2004)	-0.30	-0.54	-0.05
Menegassi (2009)	-0.43	-0.71	-0.15
Juneja (2010)	-0.30	-0.54	-0.06
Kwon (2011)	-0.44	-0.73	-0.16
Mahmoud (2011)	-0.39	-0.67	-0.11
Cortese (2012)	-0.34	-0.60	-0.08
Donfrancesco (2013)	-0.46	-0.75	-0.17
Adisetiyo (2014)	-0.47	-0.73	-0.22
Bener (2014)	-0.43	-0.79	-0.076
Percinel (2016)	-0.44	-0.75	-0.13

### Meta-regression Analysis

To explore sources of heterogeneity among studies, we performed a meta-regression analysis. This indicated that year of study, sample size, gender, mean age and mean Hb did not significantly influence the pooled estimates of serum ferritin (Table 5).

**Table 5:** Meta-regression of serum ferritin levels ADHD and controls.

Moderator	No. of comparisons	P value	95% Confidence Interval	
Publication Year	10	0.07	-0.01	0.23
Age (mean, years)	10	0.38	-0.17	0.41
Gender (% female)	10	0.33	0.22	59.2
Hb (g/dl)	8	0.10	-0.15	1.28
Sample size	9	0.73	0.99	1.00

### Publication Bias

**Fig 5:**
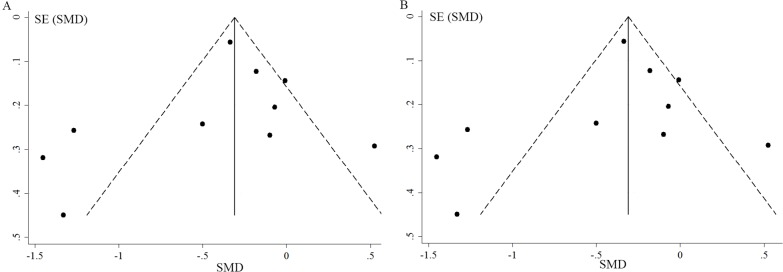
Funnel plot for publication bias test between-group meta-analysis (A) on serum ferritin levels (B) on serum iron levels.

Visual inspection of the funnel plots for included studies of serum iron and serum ferritin revealed some asymmetry (Fig 5A and 5B). However, Egger’s tests did not give significant evidence of publication bias among the included studies (P = 0.42 for serum ferritin, P = 0.35 for serum iron).

## Discussion

Our meta-analysis yielded 10 case-control studies investigating serum ferritin levels in a total of 2191 participants and 1196 ADHD cases. The pooled SMD from the 10 studies indicated that serum ferritin levels were lower in ADHD cases than those in healthy controls. Furthermore, this association remains statistically significant following sensitivity analyses. There was also no publication bias indicated by the funnel plots or Egger’s tests.

Iron, an important trace element, is implicated in many biological processes and plays a crucial role in the development of the brain [24]. Iron deficiency influences the cognitive, motor, social and emotional functions in children. [25] It has been reported that decreased iron concentration in the brain is associated with alterations in the conduction of cortical fibers, changes in serotonergic and dopaminergic systems, and in the formation of myelin [26, 27]. Serum ferritin, an intracellular protein that stores iron, is usually regarded as a reliable indicator of iron stores in body tissues, however, whether the serum ferritin is a good indicator of iron stores in the brain is debatable [28]. The level of serum ferritin is affected by inflammation and food intake [29]. Iron deficiency with or without anemia during childhood, especially in infancy, has a negative impact on cognition, behavior, and motor skills of children [30]. Compared with serum iron, serum ferritin is a more sensitive marker which can be detected at the early stage of iron deficiency even without anemia.

In this meta-analysis, we found lower serum ferritin levels in patients with ADHD than in healthy controls. Although the explicit mechanism of lower iron in ADHD is unclear, several lines of evidence may help to explain the process. First, it has been indicated that iron, a major cofactor of the tyrosine hydroxylase enzyme and a rate-limiting step in dopamine synthesis [31], is implicated in the pathophysiology of ADHD. Second, iron deficiency is associated with decreased dopamine transporter density and activity, resulting in increased extracellular dopamine, as well as reduced dopamine receptors in the striatum [32, 33]. Third, iron deficiency may result in dysfunction in the basal ganglia [34]. Forth, an imbalance between inhibitory/excitatory neurotransmitters is thought to play an important role in the pathophysiology of ADHD [35]. The γ-aminobutyric acid (GABA) is the major inhibitory neurotransmitter of the mammalian central nervous system and influence the level of iron in brain [36]. GABA levels were lower in the ADHD patients, which may lead to the reduced iron concentration in basal ganglia [37]. Fifth, lower thalamic iron levels were found in children with ADHD compared to controls detected by MRI, which indicated that decreased iron levels in thalamic may implicated the etiology of ADHD [8].

Furthermore, Konofal et al. [38] and Sever et al. [9] reported that after iron supplementation, the serum ferritin of ADHD patients increased significantly and ADHD symptoms improved. Based on these data it appears that iron supplementation may benefit ADHD; however, more studies with high quality should be conducted to confirm this effect.

Serum iron, which indicates the amount of circulating iron that is bound to transferrin, is markedly reduced in iron deficiency anemia. In this study, we failed to find any relationship between serum iron and ADHD. This may because all included studies excluded subjects with severe anemia, and so levels of Hb in ADHD cases and controls were comparable. Researchers have speculated that psychostimulants used in the treatment of ADHD may lead to appetite loss and reduced nutritional iron uptake, which may result in a reduction in serum ferritin in treated patients with ADHD [39]. However, the studies of Adisetiyo et al. [11], Menegassi et al. [20], and Calarge et al. [40] revealed comparable dietary calorie and iron intake in medicated patients with ADHD and non-medicated patients with ADHD. Our subgroup meta-analysis also showed that serum ferritin levels were not lower in the treated group of patients. However, it should be considered that the treatment subgroup had a much smaller sample size than the group without treatment, which may lead to type II error.

Comorbidities are common in patients with ADHD and Cortese et al. [8] found higher serum ferritin levels in psychiatric disorders other than ADHD. In our study, we also found that patients with ADHD alongside other psychiatric comorbidities had higher serum ferritin levels than those without psychiatric comorbidities. This difference may be explained by different dietary intake patterns and pathophysiologic changes associated with other psychiatric disorders. The choice of analytical technology also affects the tested concentration of serum ferritin. In our subgroup analysis based on assay method, lower serum ferritin was found in patients tested via ELISA.

There are several merits of our study. First, to our knowledge, this is the first comprehensive meta-analysis performed to assess the correlation between serum ferritin levels and ADHD. Second, since the level of unobserved heterogeneity appear to be high by assuming homogeneity method [41], we chose random-effects model and performed sensitivity analysis. Although there is high heterogeneity in our study, the results did not change in the sensitivity analysis, indicating the robustness of our findings.

However, several limitations of our study should be taken into consideration. First, the sample size of subjects in the selected studies was small, which may reduce the power of our analyses. More studies with larger numbers of participants should be conducted to clarify the relationship between serum ferritin and ADHD. Second, we performed publication bias tests, however, the studies included in this meta-analysis for serum ferritin or serum iron was no more than ten, which may decreased the power and tend to draw the conclusion that show larger treatment effects [42]. Third, serum ferritin may not completely reflect actual iron levels in the brain. Using MRI, Adisetiyo et al. [11] found lower levels of iron in the striatum and thalamus of mediation-naïve patients with ADHD. However, serum ferritin levels were not significantly different between mediation-naïve patients with ADHD and healthy controls. To assess brain iron levels correctly, further studies should consider combining more indices reflecting blood iron status (transferrin, total iron binding capacity) or non-invasive methods with high sensitivity and specificity, such as MRI to assess brain iron directly. Fourth, different assay techniques were used in the included studies, such as ELISA, electrochemiluminescence, and competitive binding radioimmunoassay technique. To our knowledge, there has been no comparison of different techniques. Therefore, it is not possible to exclude the possibility that differences in techniques used to measure serum ferritin values may result in variable findings [15]. Fifth, the serum iron level is affected by various factors, such as dietary intake, the disease condition, the time of draw, that were not completely considered in the included studies. Sixth, in this meta-analysis, the small number of included studies declined the power of meta-regression analyses. Although except for meta-regression analyses, we also used a random-effects model, subgroup analyses, however, we failed to identify the factors leading to heterogeneity. Therefore, the results should be interpreted cautiously.

## Conclusion

Our meta-analysis shows that serum ferritin levels are lower in patients with ADHD than in healthy controls, which suggests that serum ferritin is correlated with ADHD. In this study, we failed to find a correlation between serum iron and ADHD. This is likely due to the fact that serum iron is affected by various factors that were not completely considered in the included studies. There is a need for more high-quality studies with larger sample sizes, assessed using the same assay techniques, and multiple indices of iron status to provide more conclusive results. The mechanisms leading to iron deficiency in ADHD, and the correlation between brain iron and peripheral iron levels also needs further research.

## Supporting Information

S1 File PRISMA 2009 checklist. (DOC)
